# Clinical Predictors and Outcome of Metabolic Acidosis in Under-Five Children Admitted to an Urban Hospital in Bangladesh with Diarrhea and Pneumonia

**DOI:** 10.1371/journal.pone.0039164

**Published:** 2012-06-15

**Authors:** Mohammod J. Chisti, Tahmeed Ahmed, Hasan Ashraf, A. S. G. Faruque, Pradip K. Bardhan, Sanjoy Kumer Dey, Sayeeda Huq, Sumon Kumar Das, Mohammed A. Salam

**Affiliations:** 1 Clinical Services (CS), International Centre for Diarhoeal Disease Research, Bangladesh (icddr, b), Dhaka, Bangladesh; 2 Centre for Nutrition & Food Security (CNFS), icddr, b, Dhaka, Bangladesh; 3 Department of Neonatology, Bangabandhu Sheikh Mujib Medical University (BSMMU), Dhaka, Bangladesh; 4 Research Administration Services (RAS), icddr, b, Dhaka, Bangladesh; Aga Khan University, Pakistan

## Abstract

**Background:**

Clinical features of metabolic acidosis and pneumonia frequently overlap in young diarrheal children, resulting in differentiation from each other very difficult. However, there is no published data on the predictors of metabolic acidosis in diarrheal children also having pneumonia. Our objective was to evaluate clinical predictors of metabolic acidosis in under-five diarrheal children with radiological pneumonia, and their outcome.

**Methods:**

We prospectively enrolled all under-five children (n = 164) admitted to the Special Care Ward (SCW) of the Dhaka Hospital of icddr, b between September and December 2007 with diarrhea and radiological pneumonia who also had their total serum carbon-dioxide estimated. We compared the clinical features and outcome of children with radiological pneumonia and diarrhea with (n = 98) and without metabolic acidosis (n = 66).

**Results:**

Children with metabolic acidosis more often had higher case-fatality (16% vs. 5%, p = 0.039) compared to those without metabolic acidosis on admission. In logistic regression analysis, after adjusting for potential confounders such as age of the patient, fever on admission, and severe wasting, the independent predictors of metabolic acidosis in under-five diarrheal children having pneumonia were clinical dehydration (OR 3.57, 95% CI 1.62–7.89, p = 0.002), and low systolic blood pressure even after full rehydration (OR 1.02, 95% CI 1.01–1.04, p = 0.005). Proportions of children with cough, respiratory rate/minute, lower chest wall indrawing, nasal flaring, head nodding, grunting respiration, and cyanosis were comparable (p>0.05) among the groups.

**Conclusion and Significance:**

Under-five diarrheal children with radiological pneumonia having metabolic acidosis had frequent fatal outcome than those without acidosis. Clinical dehydration and persistent systolic hypotension even after adequate rehydration were independent clinical predictors of metabolic acidosis among the children. However, metabolic acidosis in young diarrheal children had no impact on the diagnostic clinical features of radiological pneumonia which underscores the importance of early initiation of appropriate antibiotics to combat morbidity and deaths in such population.

## Introduction

Diarrhea and pneumonia are the two leading causes of morbidity and deaths among under-five children in developing countries [1], [2]. Among the 8.8 million global deaths in under-five children in 2008, 18% and 15% occurred due to pneumonia and diarrhea, respectively [2]. Children with acute watery diarrhea (AWD) often present with dehydration which is commonly associated with metabolic acidosis [3], [4], [5] due to fecal loss of bicarbonate [6]. Clinical features of pneumonia and metabolic acidosis often overlap [3], and consequently, the differentiation of pneumonia from metabolic acidosis in children with diarrhea may be very difficult [4]. The classical features of metabolic acidosis in children with dehydrating diarrhea is fast and deep breathing [7] (often misinterpreted as lower chest wall indrawing by the health workers), which disappears after the correction of dehydration [8], [9]. However, the basis of the clinical features of pneumonia, as recommended by World Health Organization (WHO), also include fast breathing along with lower chest wall indrawing [10], which usually persist even after full rehydration in children with dehydrating diarrhea who also have pneumonia [3]. Thus, in children with dehydrating diarrhea, the diagnosis of pneumonia might be done after full rehydration. However, rehydration in children with dehydrating diarrhea for the correction of metabolic acidosis usually takes certain amount of time [9], [11], [12], which might delay the initiation of appropriate antibiotics for children who also have pneumonia potentially increasing morbidity and possibly deaths. It is thus imperative to understand the clinical predictors of metabolic acidosis in children with co-morbidity of diarrhea and radiological pneumonia, in order to initiate prompt management of pneumonia to reduce the morbidity and deaths in such children especially in resource limited settings. To our knowledge, this everyday clinical predicament for health professionals in resource limited setting has not been well addressed in medical literature.

Around 70,000 under-five children attend the Dhaka Hospital of International Centre for Diarrheal Disease Research, Bangladesh (icddr, b) with diarrhea each year, and some of them also have metabolic acidosis and concomitant pneumonia. The aim of our study was to evaluate the clinical features and outcome associated with metabolic acidosis in under-five children presenting with the co-morbidity of radiological pneumonia and diarrhea.

## Materials and Methods

### Patient enrollment

Children of both sex, aged 0–59 months, admitted to the Special Care Ward (SCW) of the Dhaka Hospital of icddr, b from September through December 2007 with co-morbidity of diarrhea and pneumonia, who had their total serum carbon-dioxide (TCO_2_) were included into the study.

Each year, this hospital provides care and treatment to over 120,000 patients of all ages. The majority of the patients come from a poor socio-economic background living in urban and peri-urban Dhaka. This being mainly a diarrhea treatment facility, essentially all patients have diarrhea with or without associated complications, but some have other health problems too. The majority of the patients are under-five children and malnutrition and pneumonia are the most common co-morbidities. On arrival to the hospital triage nurses obtain medical history and perform physical examination, and make a quick assessment of the patients, focusing on the diarrhea severity and its complications as well as diarrhea associated health problems, particularly malnutrition and pneumonia. The patients are then referred to the emergency unit where physician re-assess and admits them to an appropriate ward of the hospital. Patients with severe illnesses, including those with abnormal mental status, severe and very severe pneumonia, cyanosis, hypoxemia, suspected sepsis, and convulsions are admitted to the SCW for further assessment, closer observation, and appropriate laboratory workup and management. After admission to the SCW, attending physicians re-evaluate the patients, initiate the needed work ups, and prescribe a management plan.

### Ethics statement

Originally this data has been obtained from a prospective hospital audit which was initially designed to defend the thesis in Masters of Medicine (MMed) of the primary author in the University of Melbourne (UOM), Melbourne, Australia. Although the clinical audit, which is routine for hospital care, was done prior to the approval by the Ethical Review Committee (ERC) of icddr, b, approval was obtained from the ERC after the completion of the clinical audit to defend the thesis of the primary author under the UOM. Thus, this manuscript is the part of the approved study by the ERC of our icddr, b.

### Study design

In this study we prospectively enrolled all the children of both sex, aged 0 to 59 months, who were admitted to the SCW from September 2007 through December 2007with the co-morbidity of diarrhea and radiological pneumonia and who had their total serum carbon-dioxide (TCO_2_) measured. Comparison of the clinical features of diarrhea and pneumonia was made between the children with and without metabolic acidosis defined as total serum carbon-dioxide (TCO_2_) of less than 17 mMol/L [13]. Pneumonia was diagnosed based on radiological evidence of consolidation or patchy opacities [14]. Diarrhea was defined as the passage of three or more abnormally loose or watery stool in the previous 24 hours [15]. Relevant clinical information was collected soon after their enrollment into the study, after obtaining verbal consents from parents/attending caregivers by the attending physician.

Standard hospital guidelines were followed in the clinical management of the study patients, which included correction of dehydration using either oral rehydration salt solution (for those with some dehydration) and/or intravenous fluids (for those with severe dehydration and also those who were unable to drink due to any reason), as appropriate; antimicrobial therapy; feeding, and administration of micronutrients (vitamins and minerals) when indicated. Management of severe protein-energy malnutrition was done in accordance with the hospital's protocolised guidelines [11], [16].

### Statistical Methods

We developed and pre-tested Case Report Forms for data acquisition. All data were entered onto a personal computer and edited before analysis using SPSS for Windows (version 15.0; SPSS Inc, Chicago) and Epi Info (version 6.0, USD, Stone Mountain, GA). Differences in proportions were compared by the Chi-square test or Fisher Exact test when applicable and differences of means were compared by Student's t-test or Mann-Whitney test, as appropriate. A probability of less than 0.05 was considered statistically significant. Strength of association was determined by a calculating odds ratio (OR) and its 95% confidence intervals (CI). We have these statistics both in our univariate analyses and logistic regression. Characteristics analyzed include age, gender, poor socio-economic condition [monthly income less than 5,000 taka (US$ 75)], vomiting, clinical dehydration, breast-feeding history, fever, cough, hypoxemia, respiratory rate (counted in one minute in a calm child), lower chest wall indrawing (indrawing of the bony structures of the lower chest wall during inspiration), head nodding, nasal flaring, cyanosis, grunting respiration, abnormal mental status (restless or irritable or lethargy or disoriented), severe wasting [z score for weight for height <−3 of the WHO growth standard], systolic blood pressure and outcome. Initially, we performed univariate analyses of these characteristics to identify factors that were significantly associated with metabolic acidosis and finally, we performed logistic regression analysis of the factors significantly associated with metabolic acidosis after adjusting with potential confounders to identify the actual impact of metabolic acidosis on clinical features of pneumonia and diarrhea.

## Results

In total 164 children were enrolled in the study and 66 of them had metabolic acidosis. Children with metabolic acidosis were younger, less often were febrile at admission, more often had severe wasting and more likely to have fatal outcomes than those without metabolic acidosis (Table 1). The number of patients with low systolic blood pressure despite correction of dehydration among the diarrheal children with pneumonia and metabolic acidosis compared to those without metabolic acidosis was significantly higher (33% vs. 4%; p = 0.002) (Table 1). In logistic regression analysis, after adjusting for potential confounders, such as age of the patient, fever on admission, and severe wasting, the independent predictors for metabolic acidosis in children under five with pneumonia and diarrhea were clinical dehydration and low systolic blood pressure even after correction of dehydration (Table 2). There was no significant difference of presence of clinical features of pneumonia such as cough, respiratory rate, lower chest wall indrawing, nasal flaring, head nodding, grunting respiration, and cyanosis among the groups (Table 1). The distribution of sex, poor socio-economic condition, vomiting, non-breast-fed, abnormal mental status, and hypoxemia were not different between the groups (Table 1).

**Table 1:** Clinical characteristics of under-five children having pneumonia and diarrhea with (cases) and without metabolic acidosis (controls).

Characteristic	Cases (n = 98)	Controls (n = 66)	OR	95% CI	p value
Male sex	58 (59)	33 (50)	1.45	0.74–2.86	0.317
Age in months (median, IQR)	4.8 (2.0, 10.0)	8.5 (4.0, 14.1)	-	-	0.004
Poor socio-economic condition	60 (63)	41 (62)	1.01	0.51–2.04	0.908
Children with vomiting	75 (77)	42 (64)	1.86	0.89–3.92	0.106
Clinical dehydration (some/severe)	64 (67)	18 (2)	5.02	2.41–10.56	<0.001
Children with cough	87 (89)	59 (89)	0.94	0.31–2.81	0.896
Non-breastfed	40 (41)	19 (29)	1.67	0.81–3.45	0.180
Fever on admission (≥38°Celsius)	80 (82)	62 (94)	0.29	0.08–0.96	0.042
Respiratory rate (/minute) (mean ± SD)	57.6±19.6	58.0±16.3	-	-	0.901
Systolic blood pressure (mm of Hg) (mean ± SD)	84.3±36.2	102.2±22.5	-	-	<0.001
Low systolic blood pressure (≤70 mm of Hg) despite full correction of dehydration	30 (33)	4 (7)	7.25	2.24–25.98	0.002
Lower chest wall in drawing	69 (71)	49 (74)	0.85	0.40–1.83	0.797
Nasal flaring	25 (26)	13 (20)	1.42	0.62–3.24	0.477
Head nodding	4 (4)	2 (3)	0.91	0.16–5.32	1.00
Grunting respiration	3 (3)	4 (6)	0.49	0.08–2.72	0.441
Cyanosis	10 (10)	4 (6)	1.76	0.48–7.02	0.518
Abnormal mental status	81(83)	49 (74)	1.76	0,76–4.06	0.213
Hypoxemia (SPO2<90%)	50 (51)	36 (55)	0.87	0.44–1.70	0.777
WHZ (<−3 z score)	31 (32)	10 (16)	2.54	1.07–6.11	0.032
Outcome (Died)	16 (16)	3 (5)	4.10	1.06–18.56	0.039

Figures represent n (%), unless specified. OR: odds ratio. CI: confidence interval.

IQR: inter-quartile range.SD: standard deviation. WHZ: weight for height z score; SpO_2_ = transcutaneously measured blood oxygen concentration.

**Table 2:** Results of logistic regression to explore independent predictors for metabolic acidosis in diarrheal children with pneumonia.

Characteristics	OR	95% CI	p value
Age in months	1.01	0.98–1.05	0.418
Clinical dehydration (some/severe)	3.57	1.62–7.89	0.002
Fever on admission (°Celsius)	0.46	0.13–1.57	0.212
Severe wasting	0.84	0.30–2.32	0.733
Low systolic blood pressure despite correction of dehydration	1.02	1.01–1.04	0.005

OR: odds ratio. CI: confidence interval.

## Discussion

We observed significantly higher deaths in children with diarrhea and pneumonia and metabolic acidosis compared to those without metabolic acidosis. The children having diarrhea and pneumonia with metabolic acidosis more often had younger age and frequently presented with severe wasting compared to those without metabolic acidosis. Young infant compared to their elder counterpart, usually do not have adequate immunity, more vulnerable to severe infection, and likelihood of fatal outcome [17]. Moreover, malnutrition in children often causes depressed cell mediated and humoral immune responses, frequently associated with impairment of IgA production, inefficient chemotaxis, reduced mature T cells, and compromised phagocytic activity [18]. As a result, they become highly susceptible to severe infection, often leading to sepsis [15] and subsequent severe metabolic acidosis and death. Furthermore, significantly higher proportion of diarrheal children with pneumonia and metabolic acidosis compared to those without metabolic acidosis had clinical dehydration which is often associated with high case-fatality [19]. This observation might have an additional impact on the higher deaths in diarrheal children with pneumonia and metabolic acidosis. Thus, these explain our observation of frequent deaths in children with diarrhea and pneumonia with metabolic acidosis. Our infrequent observation of fever among the diarrheal children with pneumonia and metabolic acidosis might be due to the fact that most of the children presented with severe malnutrition which results in poor febrile response.

Our observation of more frequent low systolic blood pressure despite correction of dehydration among the diarrheal children with pneumonia and metabolic acidosis compared to those without metabolic acidosis is also understandable. Low systolic blood pressure even after correction of dehydration is one of the markers of poor peripheral perfusion, which is a feature of severe sepsis in under-five children [20], [21]. Severe sepsis is often associated with vasodilatation and capillary leakage as a result of amplified cytokines or other inflammatory stimuli [22], [23]. This leads to disordered microcirculation and increased lactate production as a by-product of anaerobic cellular respiration leading to metabolic acidosis [21].

Our observation of frequent clinical dehydration among the diarrheal children with pneumonia and metabolic acidosis compared to those without metabolic acidosis is very classical. Children with dehydrating diarrhea often have fecal loss of bicarbonate and often develop metabolic acidosis [6]. This finding is consistent with our earlier observations [3], [5].

Thus the observation of clinical dehydration and low systolic blood pressure even after correction of dehydration as independent predictors for metabolic acidosis in children under five with pneumonia and diarrhea after adjusting for potential confounders, such as age of the patient, fever on admission, and severe wasting, indicates that metabolic acidosis is a serious feature of underlying pathologies. This observation is very important and has high clinical relevance in diarrheal children with pneumonia especially in resource limited setting where there is a lack of opportunity in performing total serum carbon-dioxide. Early identification of these clinical parameters of metabolic acidosis by the health workers might help in making decision for prompt rehydration and subsequent early referral to tertiary hospitals for further and appropriate management in order to reduce morbidity and deaths in such children.

The observation of lack of influence of metabolic acidosis on cough, respiratory rate, lower chest wall indrawing, nasal flaring, head nodding, grunting respiration and cyanosis in diarrheal children with pneumonia is very important. High respiratory rate and lower chest wall indrawing are the basis of the WHO algorithm for diagnosing pneumonia in under-five children [24]. It is expected that more children with metabolic acidosis and pneumonia would be tachypnoeic. In this study population, however, a significantly higher proportion of children with diarrhea, pneumonia and metabolic acidosis presented with severe acute malnutrition compared to those without metabolic acidosis, and this may have confounded the results. The poor inflammatory response in severely malnourished children impedes an adequate rise of respiratory rate even when there is metabolic acidosis [18]. Moreover, severely malnourished children have deficient total body electrolytes (except sodium), especially potassium and magnesium [25], and this may be partly responsible for the reduced work of the respiratory accessory muscles, even in the presence of metabolic acidosis, and resulting in the lack of fast breathing and lower chest wall indrawing in such population. The observation of lack of influence of metabolic acidosis on nasal flaring and head nodding might be due to the same reason. However, the comparable distribution of cough, grunting respiration, cyanosis and hypoxemia in both the groups might be due to the fact that both the groups had pneumonia and these features are the consequence of pneumonia. The failure to find the association of sex, poor socio-economic condition, vomiting, non-breast-fed, and abnormal mental status among the groups might be due to small sample size.

Although we have underscored the importance of metabolic acidosis measured by total serum carbon-dioxide in diarrheal children with pneumonia, the actual cause of acidosis in our study cases may be mixed rather than metabolic only. All of our study children had both pneumonia and diarrhea. Acidosis in pneumonia is usually respiratory in origin [26] and on the other hand, diarrhea leads to metabolic acidosis [6]. Thus, the acidosis in our study population is likely to be mixed acidosis.

In conclusion, metabolic acidosis in under-five children having diarrhea and radiological pneumonia is associated with increased deaths compared to those without metabolic acidosis. Diarrheal children with radiological pneumonia presenting with clinical dehydration, and low systolic blood pressure even after correction of dehydration are more likely to have metabolic acidosis. Metabolic acidosis, however, has no impact on the diagnostic clinical features of radiological pneumonia in such children. It underscores the importance of early initiation of appropriate antibiotics in under-five diarrheal children with clinical features of pneumonia irrespective of the presence or absence of metabolic acidosis and simultaneously it is important to rule out severe sepsis in under-five diarrheal children with pneumonia who have metabolic acidosis, in an effort to reduce morbidity and deaths.
